# Age and sex differences in blood pressure regulation: A focus on the vascular baroreflex limb

**DOI:** 10.14814/phy2.70413

**Published:** 2025-06-19

**Authors:** Darcianne K. Watanabe, Suzi Hong, DeWayne P. Williams, Jordan Kohn, Julian Koenig, Gustavo A. Reyes del Paso, Julian F. Thayer

**Affiliations:** ^1^ School of Social Ecology University of California Irvine Irvine California USA; ^2^ Herbert Wertheim School of Public Health & Human Longevity Science; Department of Psychiatry, School of Medicine University of California San Diego La Jolla California USA; ^3^ Department of Psychological Science University of California Irvine Irvine California USA; ^4^ Department of Child and Adolescent Psychiatry, Psychosomatics and Psychotherapy, Faculty of Medicine and University Hospital Cologne University of Cologne Cologne Germany; ^5^ Department of Psychology University of Jaén Jaén Spain

**Keywords:** hemodynamics of blood pressure, hormone replacement therapy, hypertension, total peripheral resistance, vascular baroreflex sensitivity

## Abstract

Despite the vasculature's role in long‐term blood pressure (BP) regulation, limited work exists on vascular baroreflex function. This study focused on hypothesized age and sex differences in the vascular‐sympathetic baroreflex limb and explored the role of hormone replacement therapy (HRT). Resting cardiac and hemodynamic measures were recorded. Baroreflex sensitivity (BRS) and baroreflex effectiveness (BEI) were calculated for each baroreflex limb (cardiac, myocardial, and vascular). In younger adults, women had significantly lower SBP, total peripheral resistance (TPR), and vascular‐BEI than men (*r*'s > 0.245, *p*'s < 0.012). In older adults with managed‐hypertension, post‐menopausal women had significantly higher SBP, TPR, and vBEI, than men (*r*'s > 0.228, *p*'s < 0.018), while younger women had significantly higher vBRS (*r*'s > 0.199, *p*'s < 0.027) and lower TPR (*r*'s > 0.281, *p*'s < 0.002). Younger men showed significantly higher vascular‐BRS and vascular‐BEI (*r*'s > 0.318, *p*'s < 0.002), higher TPR and TPR‐variability (*r*'s > 0.314, *p* ≤ 0.003), and lower SBP (*r*'s > 0.295, *p*'s < 0.005) than older men. Compared to non‐HRT women, HRT‐women had only significantly lower SBP (*r =* 0.243, *p* = 0.035). We provide the first evidence of age and sex differences in vascular baroreflex‐mediated BP control using the sequence method.

## 1 INTRODUCTION

Hypertension and cardiovascular disease risk increase with age, particularly among post‐menopausal women (Benjamin et al., 2017; Whelton et al., 2018; Willey et al., 2014). There are known sex and age differences in blood pressure (BP) control, and the baroreflex has been implicated in BP regulation (Joyner et al., 2014, 2016; Lohmeier, 2001; Lohmeier & Iliescu, 2015; Sleight, 2004; Thayer et al., 2016; Thrasher, 2004, 2005). Estrogen/Progesterone have been implicated in the beneficial cardiovascular profile of premenopausal women (Chakrabarti et al., 2014; Lima et al., 2012; Sabbatini & Kararigas, 2020), but the role of these hormones in elevated hypertension and CVD risk observed among post‐menopausal women is less clear (Benjamin et al., 2017; Cho et al., 2023; Cutler et al., 2008; Joyner et al., 2014). Moreover, studies have not simultaneously examined these age and sex differences specifically in the vascular limb of the baroreflex using the sequence method to estimate baroreflex function. Thus, the primary goal of this paper is to elucidate the role of the vascular limb of the baroreflex on known age and sex differences in BP regulation. An exploratory aim is to examine the effect of hormone replacement therapy (HRT) with estrogen/progesterone on vascular function and BP regulation in older women.

The hemodynamics underlying BP regulation include cardiac output (CO), a measure of blood volume outputted from the heart per minute (computed as the product of heart rate and the volume of blood ejected from the heart in each beat [stroke volume] SV), and total peripheral resistance (TPR), a measure of resistance to blood flow in the vasculature (Brook & Julius, 2000; Guyton & Hall, 2000). Hypertension characterized by elevated TPR (i.e., vasoconstriction) rather than CO has been linked with an increased risk of CV events, morbidity, and mortality (Fagard et al., 1996; Mensah et al., 1993). However, despite well‐documented sex and age differences in BP control, the physiological mechanisms by which hypertension and CVD risk among women increase with age are still being investigated (Cho et al., 2020; Cutler et al., 2008; Fagard et al., 1996; McCarthy et al., 2014; Rosano et al., 2007; Whelton et al., 2018). Better BP regulation in pre‐menopausal women compared to age‐matched men (e.g., lower hypertension risk) (Benjamin et al., 2017; Cutler et al., 2008) has been linked to increased vasodilatory capacity (i.e., lower TPR), sex hormones (e.g., endogenous estrogen), endothelial function (Chakrabarti et al., 2014; Hart et al., 2014; Lavi et al., 2007; Moreau et al., 2012, 2020; Stanhewicz et al., 2018; Taddei et al., 1996) and differences in muscle sympathetic nerve activity (MSNA) (Hart et al., 2011, 2014; Keir et al., 2020; Narkiewicz et al., 2005). For instance, though findings are inconsistent (Vianna et al., 2012), MSNA burst frequency (bursts/min) tends to be lower in younger women compared to similarly aged men (Keir et al., 2020), with MSNA showing no relationship with TPR or BP in younger women and positive relationships in younger men (Hart et al., 2009, 2011, 2014). Indeed, greater reflexive BP and TPR responses to spontaneous MSNA bursts in younger men than younger women (Briant et al., 2016; Robinson et al., 2019) are consistent with the idea that the protective effects of estrogen in the vasculature may be more beneficial for younger women than men (Cattaneo et al., 2017; Harvey et al., 2020; Keir et al., 2020; Kim‐Schulze et al., 1996; Taddei et al., 1996). Conversely, resting MSNA increases with age (Harvey et al., 2020; Keir et al., 2020), with older adults showing more frequent MSNA bursts (Briant et al., 2016) that evoke blunted BP responses in both sexes (D'Souza et al., 2023; Petterson et al., 2022; Young et al., 2020; Vianna et al., 2012). Additionally, the relationships between MSNA, TPR, and BP become positive among healthy older women (≥50) and stronger compared to age‐matched men (Hart et al., 2014; Keir et al., 2020; Taddei et al., 1996). As noted by Fu and Ogoh (2019), previous studies using various baroreflex assessment techniques have produced mixed findings that varied as a function of different techniques used to evaluate baroreflex sensitivity (e.g., pharmacological [Oxford technique] vs. non‐pharmacological) (e.g., orthostatic challenge, Valsalva Maneuver), phase of menstrual cycle, age, and sex. Taken together, these results highlight the need to further examine age and sex differences in BP regulation, particularly within the vasculature.

The arterial baroreflex is a significant determinant of short‐term BP regulation, and increasing evidence suggests that its role in long‐term BP regulation is critical (Hesse et al., 2007; Lohmeier, 2001; Lohmeier et al., 2004; Parati et al., 2016; Sleight, 2004; Thrasher, 2002, 2004, 2005). Baroreceptors serve as an intermediary relay between the cardiovascular and central nervous systems via stretch‐pressure sensitive afferent fibers that regulate BP primarily via the control of CO and TPR (Benarroch, 2008; James, 1971; Kumada et al., 1990). These receptors are primarily under the control of the parasympathetic nervous system. Thus, an increase in BP elicits parasympathetic activation and sympathetic inhibition, resulting in reflexive responses by each baroreceptor limb, which serve to regulate beat‐to‐beat BP (i.e., baroreflex sensitivity) (Benarroch, 2008; Duschek & Reyes del Paso, 2007; Heymans, 1960; Reyes Del Paso et al., 1996). Namely, the cardiac limb modulates interbeat intervals (IBI); the myocardial limb, contractility (SV); and the vascular limb, blood vessel diameter (i.e., vasomotor tone; TPR). As such, a disproportionate number of BP changes uncoupled with the expected reflexive modulation may indicate baroreflex ineffectiveness (Di Rienzo et al., 2001). While a complete discussion about the various methods used to measure baroreflex function is beyond the scope of this paper (Parati et al., 2001), it is important to note that most studies have focused on the cardiac and myocardial baroreflex limbs or used an indirect measure of the vascular limb (Christou et al., 2005; Vaschillo et al., 2011; Yasumasu et al., 2005). Moreover, although vascular function plays a critical role in long‐term blood pressure regulation and there is substantial evidence of age and sex differences in cardiovascular outcomes, the vascular baroreflex limb has not explicitly been studied using the sequence method to estimate baroreflex function (Christou et al., 2005; Hesse et al., 2007; Parati et al., 2016; Thrasher, 2002, 2004). Additionally, age and sex‐related differences across all three limbs of the baroreflex remain unexplored.

Thus, we address the gap in the literature on vascular baroreflex function by documenting age and sex differences in blood pressure regulation, focusing on the vascular limb of the baroreflex using the sequence method. We hypothesized that, consistent with prior work focusing on the cardiac baroreflex limb, vascular baroreflex control of BP would also vary by age and sex (Chapleau et al., 1995; Gribbin et al., 1971; Monahan et al., 2001; Schumann et al., 2024). As mixed findings exist regarding the protective effects of HRT (e.g., exogenous estrogen and/or progesterone) on BP, in the vasculature, and CVD risk (Cho et al., 2023; Gu et al., 2024; Hodis et al., 2016; Novella et al., 2012; Rossouw et al., 2002; Saitta et al., 2001), exploratory analyses were conducted to examine the potential protective role of HRT on BP control. An examination of age and sex differences in baroreflex function is highly relevant and clinically significant, as baroreflex function has been linked to BP control, the etiology of hypertension and cardiovascular outcomes (Bertinieri et al., 1988; Billman et al., 1982; Duschek & Reyes del Paso, 2007; La Rovere et al., 2008; Lohmeier, 2001; Lohmeier & Iliescu, 2015; Sleight, 1997, 2004), and it is well understood that these outcomes vary by age and sex (Benjamin et al., 2017; Cho et al., 2020; Kerola et al., 2021; Millett et al., 2018; Whelton et al., 2018; Willey et al., 2014).

## 2 METHODS

Beat‐to‐beat systolic blood pressure (SBP) and heart rate (HR) were recorded from the non‐dominant middle finger by using the Finometer Pro, which uses the Modelflow three‐element Windkessel method to estimate CO and produces values comparable to those obtained via thermodilution (Finapres Measurement Systems, Amsterdam, The Netherlands) (Jansen et al., 2001; Sollers et al., 2006) and high linear correlations and tolerable bias with ECG‐derived measures (Del Reyes Paso et al., 2010). Compared to traditional plethysmograph measures, the Finapres signal is a fairly stable signal, with fewer artifacts and less vasomotor influence (Imholz et al., 1998). As we have done in prior work, we used the sequence method and custom‐made LabVIEW software (LabVIEW8.5, National Instruments, Austin, Texas, USA) to calculate cardiac‐vagal, vascular‐sympathetic, and myocardial spontaneous baroreflex function in the time domain (Di Rienzo et al., 2001; Duschek & Reyes del Paso, 2007; Reyes et al., 2004; Reyes del Paso et al., 2006, 2017, 2024). The sequence method, as instantiated in this custom‐made software, enables us to simultaneously estimate baroreflex function in each baroreflex limb (cardiac, vascular, and myocardial) (Reyes Del Paso et al., 1996). The custom program derives mean indices of IBIs, TPR, SBP, and SV. Variability in TPR, SBP, and SV was calculated as the standard deviation (e.g., TPRv) and IBIs as HRV (root mean square of successive differences [RMSSD]). As Marchi et al. (2016) noted, the sequence method allows for a common framework for the contemporaneous assessment of various branches of the baroreflex. Therefore, as suggested by Marchi et al. (2016), we utilized the sequence method and further discriminated the three limbs of the baroreflex (Reyes del Paso et al., 2017), which therefore overcomes many of the shortcomings associated with other methods of baroreflex assessment.

To distinguish the three baroreflex branches and maintain a common framework (Marchi et al., 2016), SBP was used as a probe to assess resting baroreflex sensitivity (BRS) in each baroreflex limb (cardiac, vascular, and myocardial) using the appropriate lags (Reyes del Paso et al., 2017, 2024). BRS is expressed as the magnitude of the average beat‐to‐beat change in each output measure, IBI (ms), TPR (dyn/s/cm^5^), or SV (mL/beat), per 1 mmHg SBP in each sequence. The cardiac‐vagal BRS was determined as a slope of the linear regression between the SBP changes (in mmHg) and the subsequent IBI changes (in ms) for all sequences where SBP and IBI values either increased (up‐sequences) or decreased (down‐sequences) within 3–6 consecutive beats and with zero delay (i.e., SBP/IBI sequence) (Parati et al., 2000). These cardiac baroreflex‐mediated sequences had at least a 1 mmHg SBP change coupled with a 5 ms IBI change between beats with a correlation coefficient >0.85 (Parati et al., 2000). Vascular‐sympathetic and myocardial BRS were determined as the slope of the linear regression between the SBP ramp and the respective reflexive change. Consecutive vascular sequences had at least a 1 mmHg SBP change (i.e., SBP ramp) followed by a change in TPR of at least a 1 dyn s/cm^5^/beat with 3 beats lag (i.e., ramps). Myocardial sequences had an SBP ramp followed by at least a 0.4 mL change in SV with 2 beats lag (Reyes del Paso et al., 2017). Higher absolute vascular and myocardial sensitivity values indicate higher sensitivity in each limb. The extreme and artifact values for the IBI and TPR (80% > IBI > 120%) and for the SV (90% > IBI > 110%), compared to several previous beats, were rejected. The baroreflex effectiveness index (BEI), which indexes spontaneous SBP changes that are followed by the expected reflexive modulation (Duschek & Reyes del Paso, 2007), was calculated as a ratio of the total number of sequences (i.e., SBP/IBI, SBP/TPR, SBP/SV) to the total number of uncoupled SBP ramps and expressed as a percent (Di Rienzo et al., 2001; Reyes del Paso et al., 2017).

BRS is expressed as the magnitude of the average beat‐to‐beat change in each output measure, IBI (ms), TPR (dyn/s/cm^5^), or SV (mL/beat), per 1 mmHg SBP in each sequence. The cardiac‐vagal BRS was determined as a slope of the linear regression between the SBP changes (in mmHg) and the subsequent IBI changes (in ms) for all sequences where SBP and IBI values either increased (up‐sequences) or decreased (down‐sequences) within 3–6 consecutive beats and with zero delay (i.e., SBP/IBI sequence) (Parati et al., 2000). These cardiac baroreflex‐mediated sequences had at least a 1 mmHg SBP change coupled with a 5 ms IBI change between beats with a correlation coefficient greater than 0.85 (Parati et al., 2000). Vascular‐sympathetic and myocardial BRS were determined as the slope of the linear regression between the SBP ramp and the respective reflexive change. Consecutive vascular sequences had at least a 1 mmHg SBP change (i.e., SBP ramp) followed by a change in TPR of at least a 1 dyn s/cm^5^/beat with a 3 beats lag (i.e., ramps). Myocardial sequences had an SBP ramp followed by at least a 0.4 mL change in SV with a 2 beats lag (Reyes del Paso et al., 2017). Higher absolute vascular and myocardial sensitivity values indicate higher sensitivity in each limb. The extreme and artifact values for the IBI and TPR (80% > IBI > 120%) and for the SV (90% > IBI > 110%), compared to several previous beats, were rejected. The baroreflex effectiveness index (BEI), which indexes spontaneous SBP changes that are followed by the expected reflexive modulation (Duschek & Reyes del Paso, 2007), was calculated as a ratio of the total number of sequences (i.e., SBP/IBI, SBP/TPR, SBP/SV) to the total number of uncoupled SBP ramps, and expressed as a percent (Di Rienzo et al., 2001; Reyes del Paso et al., 2017).

As BRS and BEI were calculated for each branch of the baroreflex, cardiac BRS and BEI are referred to as cBRS and cBEI, vascular as vBRS and vBEI, and myocardial as mBRS and mBEI. The number of beats between changes in SBP and output measures (beat lags, e.g., TPR) was considered for both BRS and BEI and varied by baroreceptor branch. Due to differences in time lags of the cardiac, vascular, and myocardial responses, beat lags 0 and 1 were considered for the cardiac branch, beat lags 3–6 for the vascular branch, and beat lags 1–3 for the myocardial branch (see (Reyes del Paso et al., 2017), for further details regarding beat lag selection).

### 2.1 Sample one–younger adults

For the first sample, data were available from 106 individuals (48 women, 58 men; *M*_age_ = 20.1 [3.7]). Demographic variables, including sex, ethnicity, height, weight, and age, were collected. Participants were seated upright in a chair for a minimum of 10 min rest prior to the first baseline blood pressure measurement. The spontaneous baroreflex testing was performed for 5 min in a resting sitting position. Experiments were conducted between 9:00 am and 5:00 pm in a quiet, dimmed room. Participants were to refrain from exercise, caffeine, and smoking 6 h before the experiment. The Ohio State University Institutional Review Board approved all procedures, and the study was performed according to the ethical standards of the 1964 Declaration of Helsinki. Written informed consent was obtained from all participants.

### 2.2 Sample two–older adults

For the second sample, data were available from 109 individuals with managed hypertension, defined as hypertensive individuals taking medication under the ongoing care of a physician (76 women [17 on HRT], 33 men; *M*_age_ = 72.9 [8.5]). To provide meaningful comparisons with the normotensive adults in sample one, adults with managed hypertension were included in sample two. As such, approximately 62.4% (68 of 109) of the sample were taking at least one cardiovascular medication. Demographic variables, including sex, ethnicity, height, weight, and age, were collected. Of the 65/73 (89%) who reported their last menstrual period, all were post‐menopausal. The eight women who failed to report their last menstrual period had a mean age of 77.6 years (range: 67.1–93.3 years), suggesting that, given the average age of menopause is ≈51 (Zhu et al., 2018), they too were post‐menopausal. Participants were seated upright in a chair for a minimum of 10 min rest prior to the first baseline blood pressure measurement. The spontaneous baroreflex testing was performed for 5 min in a supine resting position. Experiments were conducted in the early afternoon within a quiet, dimmed room, at 21°C and relative humidity of 50%–70% after refraining from exercise, caffeine, smoking, and vitamins for 12 h. The University of California, San Diego Institutional Review Board approved all procedures, and the study was performed according to the ethical standards of the 1964 Declaration of Helsinki. Written informed consent was obtained from all participants. As described above in sample one, beat‐to‐beat SBP and HR were recorded using the Finometer Pro, which was calibrated using an automated brachial cuff at every session (Del Reyes Paso et al., 2010; Imholz et al., 1998; Jansen et al., 2001; Sollers et al., 2006). Similarly, an identical sequence method and LABVIEW software were used to assess mean and variability measures, as well as BRS and BEI for the cardiac, vascular, and myocardial limbs of the baroreflex (Di Rienzo et al., 2001; Duschek & Reyes del Paso, 2007; Reyes et al., 2004, 2006, 2017, 2024).

## 3 STATISTICAL ANALYSIS

All statistical tests were conducted using SPSS (ver. 20, IBM, Chicago, IL, USA). All tests were two‐tailed, and significance levels were evaluated using an alpha of 0.05.

We first stratified subjects into groups based on their reported sex (men and women). Physiological data were carefully reviewed for extreme statistical outliers. All participants were retained as their values were deemed physiologically plausible, ensuring the inclusivity and representativeness of our sample. Independent samples *t*‐tests were used to determine sex differences in both samples' resting physiological and baroreflex variables (all beat lags). *T*‐values, effect sizes (*r*), and *p*‐values are reported. For both samples, all difference tests statistically controlled for age, ethnicity (dummy coded), and body mass index (BMI), and results were identical when considering these covariates. Thus, *t*‐tests without covariates are reported.

Univariate Analysis of Variance (ANOVA) tests were conducted to examine group differences in baseline physiological and baroreflex variables between men and women with and without HRT. Preplanned contrasts (*t*‐tests) were used to determine planned differences between groups.

## 4 RESULTS

### 4.1 Sample one–younger adults

#### 4.1.1 Sample baseline characteristics

The average age for the total sample was 19.99 years, with men's average age of 20.10 (SD = 4.79) and women's average age of 19.86 (SD = 3.20 years). Women had an average BMI of 24.31 (SD = 4.41), while men had an average BMI of 24.80 (SD = 4.17). There were no significant differences between sexes in age (*t* (132) = 0.34, *r =* 0.029, *p* = 0.735) or BMI (*t* (130) = 0.64, *r =* 0.056, *p* = 0.523).

#### 4.1.2 Sex differences in all cardiovascular variables

Means and standard deviations for all variables stratified by sex in younger adults are presented in Table 1A. Compared to men, women showed significantly lower IBI (*t* (104) = −3.05, *r =* 0.287, *p* = 0.003), SBP (*t* (104) = −3.63, *r =* 0.335, *p* < 0.001), SBPv (*t* (104) = −3.44, *r =* 0.320, *p* = 0.001), TPR (*t* (103) = −4.01, *r =* 0.367, *p* < 0.001), and TPRv (*t* (103) = −2.98, *r =* 0.281, *p* = 0.004). Women showed significantly higher SVv (*t* (104) = 2.86, *r =* 0.270, *p* = 0.005) compared to men. There were no significant differences in HRV (*t* (103) = 0.14, *r* = 0.011, *p* = 0.909) or SV (*t* (104) = 1.63, *r* = 0.158, *p* = 0.105). Results remained the same when controlling for age, ethnicity, and BMI.

**TABLE 1:** Means and standard deviations for all cardiovascular variables stratified by age and gender.

	A: Younger individuals				*p*	B: Older individuals				*p*	C: Age comparisons	
	Women		Men			Women		Men			*p*‐_women_	*p*‐_men_
	Mean	SD	Mean	SD		Mean	SD	Mean	SD			
IBI (ms)	780.45	130.02	855.67	123.21	0.**003**	945.3	130.59	945.12	149.37	0.995	**<0.001**	0.**003**
HRV (ms)	51.05	27.37	51.63	24.82	0.909	31.81	24.8	34.09	27.32	0.673	**<0.001**	0.**003**
SBP (mmHg)	116.57	17.23	128.23	15.82	**<0.001**	155.29	20.58	138.3	15.68	**<0.001**	**<0.001**	0.**005**
SBPv	3.7	1.49	5.06	2.40	0.**001**	4.87	2.89	4.72	2.98	0.809	0.**010**	0.560
TPR (dyn/s/cm^5^)	821.15	228.66	1287.23	776.68	**<0.001**	1091.29	549.88	836.79	357.34	0.**018**	0.**002**	0.**003**
TPRv	53.57	30.09	79.27	52.90	0.**004**	44.87	59.85	32.3	19.65	0.249	0.352	**<0.001**
SV (mL/beat)	80.73	20.43	73.08	26.60	0.105	102.33	39.63	117.8	36.77	0.061	0.**001**	**<0.001**
SVv	4.48	1.7	3.61	1.45	0.**005**	3.16	1.9	3.8	2.09	0.124	**<0.001**	0.625
cBRS (ms/mmHg)	17.08	9.94	15.47	8.41	0.369	7.73	5.12	8.32	4.77	0.582	**<0.001**	**<0.001**
cBEI	64.63	13.19	61.12	10.63	0.132	49.97	16.98	40.17	17.65	0.**008**	**<0.001**	**<0.001**
vBRS (dyn/s/cm^5^/mmHg)	−15.18	13.47	−18.74	12.53	0.164	−9.86	12.49	−6.64	3.29	0.161	0.**027**	**<0.001**
vBEI	26.05	9.12	31.54	12.23	0.**012**	25.61	12.72	18.45	7.72	0.**004**	0.836	**<0.001**
mBRS (mL/beat/mmHg)	−1.91	0.75	−1.33	0.65	**<0.001**	−0.94	1.47	−0.93	0.43	0.951	**<0.001**	0.**002**
mBEI	41.89	13.86	35.53	13.07	0.**017**	11.60	7.88	13.88	6.74	0.158	**< 0.001**	**< 0.001**

*Note*: This table (1A: younger individuals; 1B: older individuals) depicts means and standard deviations stratified by gender for all variables of interest, including sensitivity (BRS) and effectiveness (BEI in %) for the three baroreflex branches (cardiac = c; vascula*r =* v; myocardial = m). (1C) shows *p*‐values indicating whether mean cardiovascular variables differed as a function of age within gender. Higher mBRS and vBRS absolute values represent higher BRS. Sample sizes varied based on the variable (younger women *n* = 48, younger men n's from 57 to 58; older women *n*'s from 71 to 76, older men *n*'s from 31 to 32). Significant differences are bolded.

Abbreviations: HRV, heart rate variability, in ms; IBI, inter‐beat intervals in milliseconds (ms); SBP, systolic blood pressure in millimeters of mercury (mmHg); SBPv, systolic blood pressure variability; SV, stroke volume in milliliters per beat (mL/beat); SVv = stroke volume variability; TPR, total peripheral resistance in dynes per second per centimeter cubed (dyn/s/cm^5^); TPRv, total peripheral resistance variability.

Women showed lesser vBEI (*t* (103) = 2.57, *r =* 0.245, *p* = 0.012), mBRS (*t* (104) = 4.25, *r =* 0.385, *p* < 0.001), and mBEI (*t* (104) = 2.43, *r =* 0.232, *p* = 0.017) relative to men. Results remained the same when controlling for age, ethnicity, and BMI.

### 4.2 Sample two–older adults

#### 4.2.1 Sample baseline characteristics

The mean age for older individuals was 72.86 (SD = 8.54) years, with men's average age of 71.76 years (SD = 7.15) and women's average age of 73.34 years (SD = 9.08). Women had an average BMI of 28.21 (SD = 6.32), and men showed an average BMI of 29.60 (SD = 5.63). There were no significant differences in age (*t* (107) = −0.89, *r =* 0.085, *p* = 0.376) or BMI (*t* (107) = 1.06, *r =* 0.101, *p* = 0.280) between the sexes.

#### 4.2.2 Sex differences in all cardiovascular variables

**FIGURE 1:**
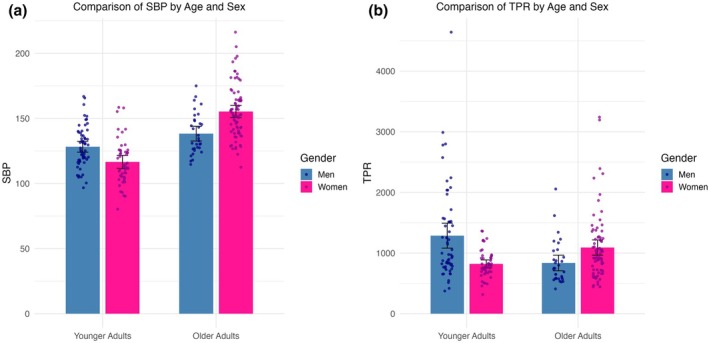
Depict mean differences between younger and older adults in systolic blood pressure in millimeters of mercury (SBP; a) and total peripheral resistance in dynes per second per centimeter cubed (TPR; b) stratified by sex, with standard error bars.

Means and standard deviations for all variables stratified by sex in older adults are presented in Table 1b. Women showed significantly higher resting SBP (*t* (106) = 4.18, *r =* 0.376, *p* < 0.001) and TPR (*t* (106) = 2.41, *r =* 0.228, *p* = 0.018) compared to men. Trending towards significance, women had lower resting SV compared to men (*t* (106) = −1.89, *r =* 0.181, *p* = 0.061). Results remained the same when controlling for age, ethnicity, and BMI (Figure 1).

Women showed significantly greater vBEI (*t* (101) = −2.91, *r =* 0.275, *p* = 0.004) and cBEI (*t* (104) = −2.70, *r =* 0.254, *p* = 0.008) compared to men. Results remained the same when controlling for age, ethnicity, and BMI.

#### 4.2.3 Age differences in all cardiovascular variables stratified by sex

Means and standard deviations are presented in Tables 1A,B, and corresponding *p*‐values denoting statistically significant differences between age groups within gender are presented in Table 1C.

**FIGURE 2:**
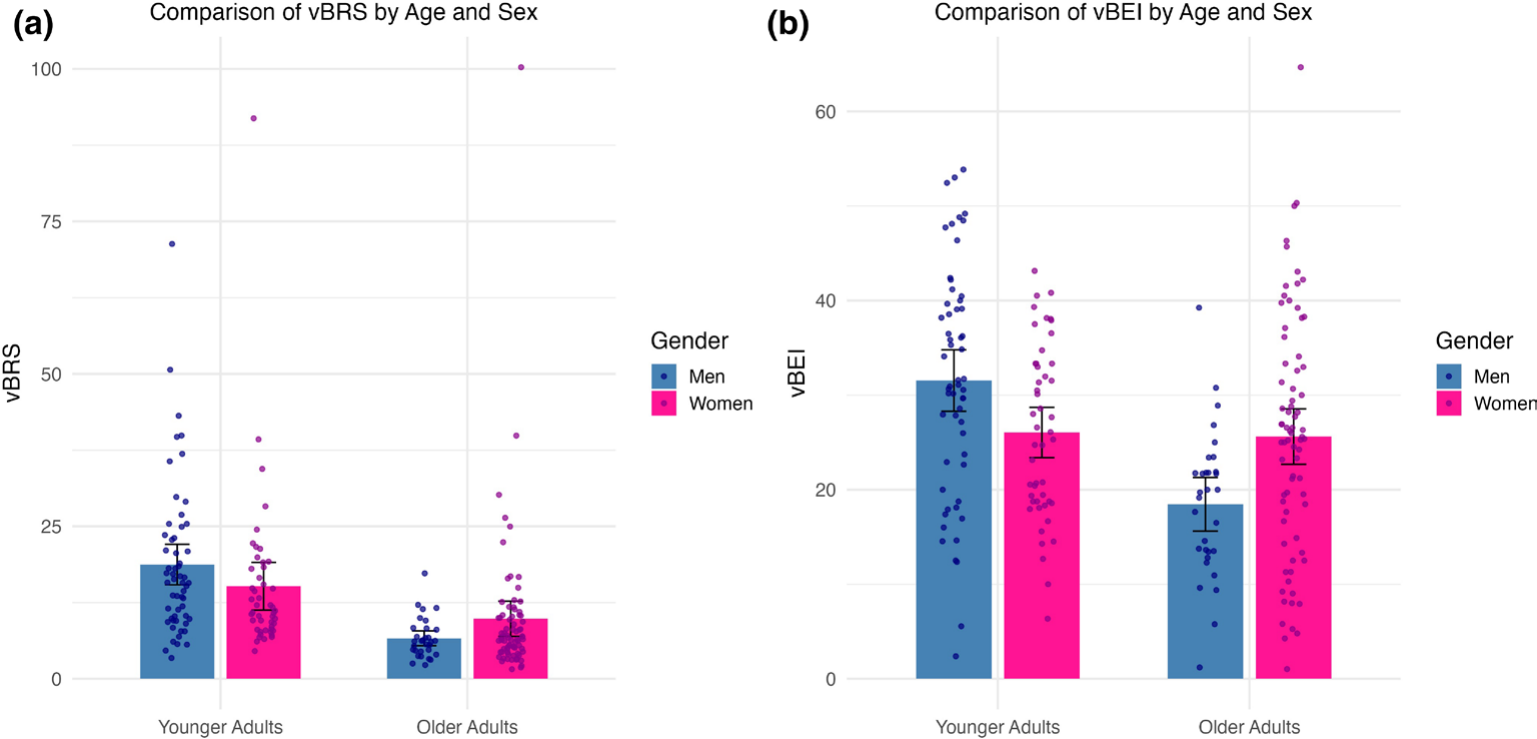
Depict mean differences between younger and older adults in baroreflex sensitivity in the vascular baroreflex limb in dynes per second per centimeter cubed per millimeters of mercury (vBRS; a) and baroreflex effectiveness in the vascular baroreflex limb in % (vBEI; b) stratified by sex, with standard error bars.

Younger women had higher HRV (*t* (122) = 4.04, *r =* 0.344, *p* < 0.001), SVv (*t* (122) = 3.95, *r =* 0.337, *p* < 0.001), cBRS (*t* (122) = 6.89, *r =* 0.529, *p* < 0.001), cBEI (*t* (122) = 5.09, *r =* 0.418, *p* < 0.001), vBRS (*t* (122) = 2.24, *r =* 0.199, *p* = 0.027), mBRS (*t* (122) = 4.18, *r =* 0.360, *p* < 0.001), and mBEI (*t* (122) = 15.30, *r =* 0.814, *p <* 0.001) compared to older women. Younger women, compared to older women, also had lower IBI (*t* (122) = −6.86, *r =* 0.528, *p* < 0.001), SBP (*t* (122) = −10.85, *r =* 0.701, *p* < 0.001), SBPv (*t* (122) = −2.61, *r =* 0.230, *p* = 0.010), TPR (*t* (122) = −3.23, *r =* 0.281, *p* = 0.002), and SV (*t* (122) = −3.49, *r =* 0.301, *p* = 0.001) (Figure 2).

**FIGURE 3:**
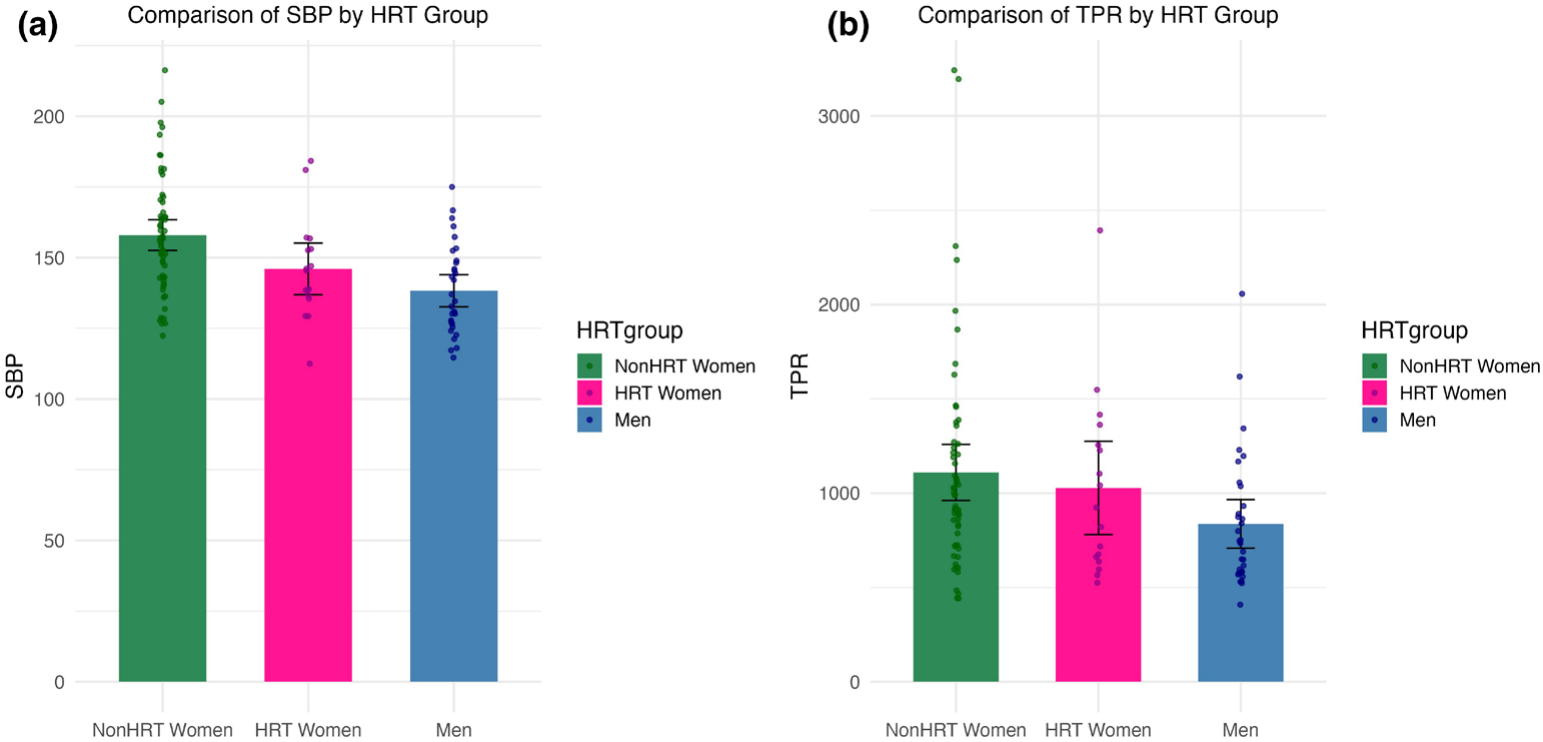
Depict mean differences between women not on HRT (NonHRT women), women on HRT (HRT women), and older men in systolic blood pressure in millimeters of mercury (SBP; a) and total peripheral resistance dynes per second per centimeter cubed per mmHg (TPR; b), with standard error bars.

Younger men had higher HRV (*t* (89) = 3.10 *r =* 0.309, *p* = 0.003), TPR (*t* (89) = 3.10, *r =* 0.315, *p* = 0.003), TPRv (*t* (89) = 4.83, *r =* 0.460, *p* < 0.001), cBRS (*t* (89) = 4.43, *r =* 0.427, *p* < 0.001), cBEI (*t* (89) = 7.03, *r =* 0.600, *p* < 0.001), vBRS (*t* (89) = 5.27, *r =* 0.494, *p* < 0.001), vBEI (*t* (89) = 5.39, *r =* 0.503, *p* < 0.001), mBRS (*t* (89) = 3.15, *r =* 0.318, *p* = 0.002), and mBEI (*t* (89) = 8.74, *r =* 0.682, *p* < 0.001) compared to older men. Younger men also had lower IBI (*t* (89) = −3.05, *r =* 0.309, *p* = 0.003), SBP (*t* (89) = −2.90, *r =* 0.295, *p* = 0.005), SV (*t* (122) = −6.64, *r =* 0.578, *p* < 0.001) compared to older men (Figure 3).

#### 4.2.4 Hormone replacement therapy exploratory analyses in older individuals

Means, standard deviations, and corresponding *p*‐values denoting statistically significant differences between groups are presented in Table 2a,b.

**TABLE 2:** Descriptive statistics for all cardiovascular variables stratified by HRT group and gender.

A: HRT in women						A: HRT sex differences					
	HRT women		Non‐HRT women		*p*		HRT women		Older men		*p*
	Mean	SD	Mean	SD			Mean	SD	Mean	SD	
IBI (ms)	909.68	111.03	955.56	134.81	0.204	IBI (ms)	909.68	111.03	945.12	149.37	0.197
HRV (ms)	29.98	36.59	32.33	20.60	0.733	HRV (ms)	29.98	36.59	34.09	27.32	0.330
SBP (mmHg)	146.04	17.77	157.95	20.70	0.**035**	SBP (mmHg)	146.04	17.77	138.30	15.68	0.062
SBPv	4.72	3.40	4.92	2.76	0.803	SBPv	4.72	3.40	4.72	2.98	0.497
TPR (dyn/s/cm^5^)	1027.67	481.37	1109.62	570.56	0.592	TPR (dyn/s/cm^5^)	1027.67	481.37	836.79	357.34	0.061
TPRv	39.70	61.11	46.36	59.93	0.689	TPRv	39.70	61.11	32.30	19.65	0.265
SV (mL/beat)	101.11	39.92	102.68	39.88	0.886	SV (mL/beat)	101.11	39.92	117.80	36.77	0.074
SVv	2.89	1.95	3.23	1.89	0.514	SVv	2.89	1.95	3.80	2.09	0.073
cBRS (ms/mmHg)	7.71	5.75	7.74	4.97	0.980	cBRS (ms/mmHg)	7.71	5.75	8.32	4.77	0.346
cBEI	43.50	17.96	51.84	16.37	0.074	cBEI	43.50	17.96	40.17	17.65	0.268
vBRS (dyn/s/cm^5^/mmHg)	−9.09	9.47	−10.08	13.31	0.776	vBRS (dyn/s/cm^5^/mmHg)	−9.09	9.47	−6.64	3.29	0.098
vBEI	23.71	16.02	26.16	11.69	0.489	vBEI	23.71	16.02	18.45	7.72	0.065
mBRS (mL/beat/mmHg)	−0.85	0.58	−0.97	1.66	0.768	mBRS (mL/beat/mmHg)	−0.85	0.58	−0.93	0.43	0.302
mBEI	11.49	7.64	11.63	8.01	0.949	mBEI	11.49	7.64	13.88	6.74	0.137

*Note*: This table (1A: HRT in women; 1B: HRT sex differences) depicts means and standard deviations stratified by HRT group for all variables of interest, including sensitivity (BRS) and effectiveness (BEI; in %) for the three baroreflex limbs (cardiac = c; vascula*r =* v; myocardial = m). (1C) shows *p*‐values indicating whether mean cardiovascular variables differed as a function of age within gender. Higher mBRS and vBRS absolute values represent higher BRS. Sample sizes varied based on the variable (HRT women *n*'s from 16 to 17, non‐HRT women *n*'s from 54 to 59, older men *n*'s from 31 to 32). Significant differences are bolded.

Abbreviations: HRV, heart rate variability, in ms; IBI, inter‐beat intervals in milliseconds (ms); SBP, systolic blood pressure in millimeters of mercury (mmHg); SBPv, systolic blood pressure variability; SV, stroke volume in milliliters per beat (mL/beat); SVv, stroke volume variability; TPR, total peripheral resistance in dynes per second per centimeter cubed (dyn/s/cm^5^); TPRv, total peripheral resistance variability.

**FIGURE 4:**
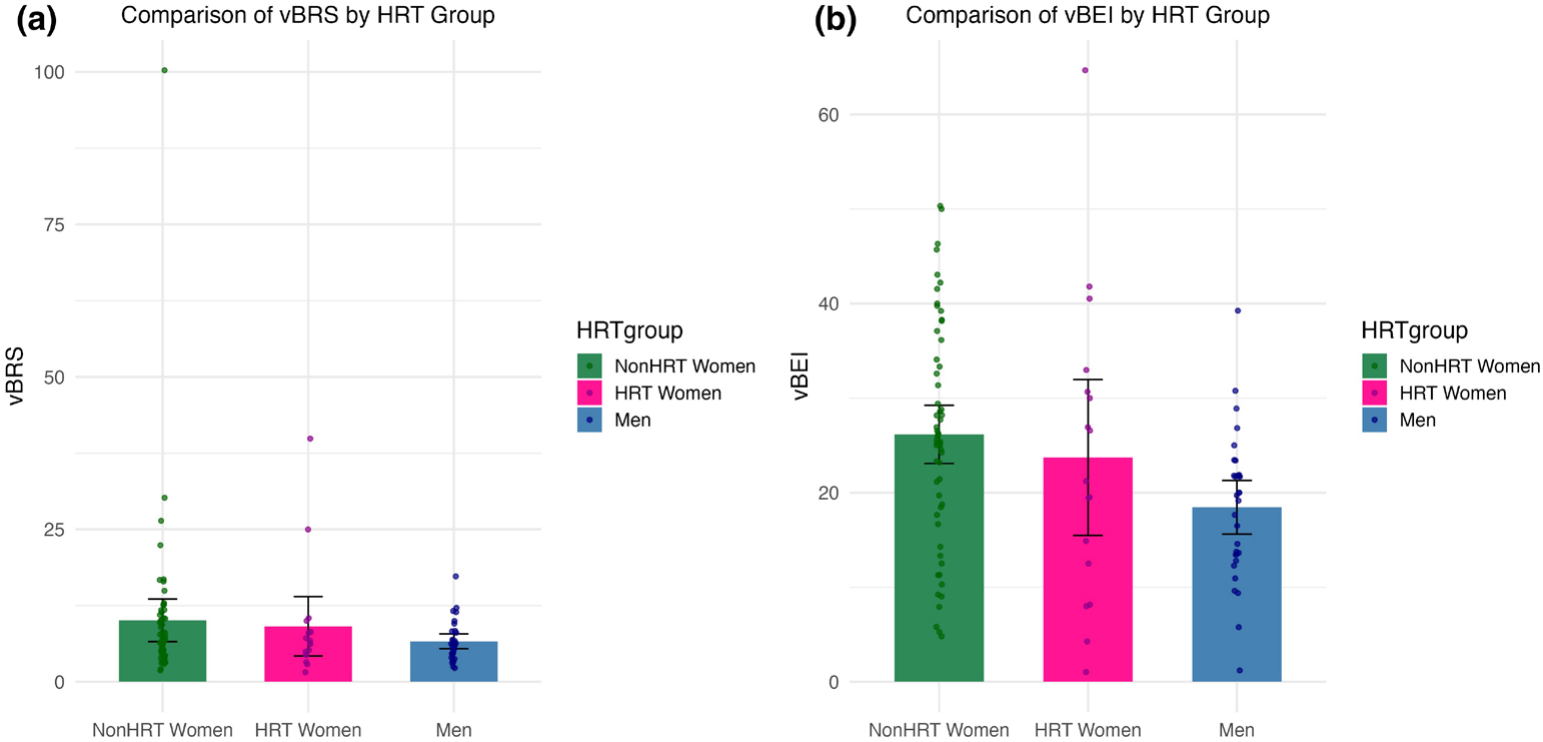
Depict mean differences between women not on HRT (NonHRT women), women on HRT (HRT women), and older men in baroreflex sensitivity in the vascular baroreflex limb in dynes per second per centimeter cubed per millimeters of mercury (vBRS; a) and baroreflex effectiveness in the vascular baroreflex limb in % (vBEI; b), with standard error bars.

NonHRT‐women had significantly higher SBP compared to both HRT‐women (*F* (1, 105) = 5.24, *r =* 0.22, *p* = 0.024) and men (*F* (1, 105) = 22.42, *r =* 0.42, *p* < 0.001); men and HRT‐women did not differ significantly in SBP (*F* (1, 105) = 1.86, *r =* 0.13, *p* = 0.175). Trending towards significance, women had lower resting SV compared to men (*t* (106) = −1.89, *r* = 0.181, p = 0.061). NonHRT‐women had significantly higher TPR (*F* (1, 105) = 6.10, *r* = 0.24, *p* = 0.015) and showed a trend towards lower SV (*F* (1, 105) = 3.14, *r* = 0.02, *p* = 0.079) compared to men. NonHRT‐women had significantly higher cBEI compared to men (*F* (1, 105) = 9.97, *r* = 0.10, *p* = 0.002) and showed a trend towards higher cBEI relative to HRT‐women (trending: *F* (1, 105) = 3.28, *r* = 0.10, *p* = 0.074) (Figure 4).

NonHRT‐women had higher cBEI compared to HRT‐women (trending: *F* (1, 105) = 3.28, *r =* 0.10, *p* = 0.074) and men (*F* (1, 105) = 9.97, *r =* 0.10, *p* = 0.002). NonHRT‐women had higher vBEI compared to men (*F* (1, 103) = 10.90, *r =* 0.11, *p* = 0.001). No other significant differences were found between groups on baroreflex sensitivity or effectiveness.

Younger women had significantly lower IBI (*t* [105] = −6.79, *r* = −0.55 [−0.74, −0.36], *p* < 0.001), SBP (*t* [105] = −11.08, *r* = −0.73 [−0.93, −0.54], *p* < 0.001), SBPv (*t* [105] = −2.76, *r* = −0.26 [−0.45, −0.07], *p* = 0.007), TPR (*t* [105] = −3.29, *r* = −0.31 [−0.50, −0.11], *p* = 0.001), and SV (*t* [105] = −3.46, *r* = −0.32 [−0.51, −0.13], *p* = 0.001), and higher HRV (*t* [105] = 4.04, *r* = 0.37 [0.18, 0.56], *p* < 0.001), SVv (*t* [105] = 3.56, *r* = 0.33 [0.14, 0.52], *p* < 0.001), cBRS (*t* [105] = 6.32, *r* = 0.52 [0.33, 0.72], *p* < 0.001), cBEI (*t* [105] = 4.38, *r* = 0.39 [0.20, 0.58], *p* < 0.001), mBRS (*t* [100] = 3.59, *r* = 0.34 [0.14, 0.53], *p* < 0.001), and mBEI (*t* [103] = 13.95, *r* = 0.81 [0.62, 1.00], *p* < 0.001) compared to nonHRT women.

Compared to HRT‐women, younger women had significantly lower IBI (*t* [63] = −3.65, *r* = −0.42 [−0.66, −0.17], *p* < 0.001), SBP (*t* [63] = −6.01, *r* = −0.60 [−0.85, −0.36], *p* < 0.001), TPR (*t* [63] = −2.34, *r* = −0.28 [−0.53, −0.04], *p* = 0.023), and SV (*t* [63] = −2.70, *r* = −0.32 [−0.57, −0.07], *p* = 0.009), and higher HRV (*t* [63] = 2.49, *r* = 0.30 [0.05, 0.55], *p* = 0.015), SVv (*t* [63] = 3.20, *r* = 0.37 [0.13, 0.62], *p* = 0.002), cBRS (*t* [63] = 3.67, *r* = 0.42 [0.17, 0.67], *p* = <0.001), cBEI (*t* [63] = 5.15, *r* = 0.54 [0.30, 0.79], *p* < 0.001), mBRS (*t* [63] = 5.25, *r* = 0.55 [0.30, 0.80], *p* < 0.001), and mBEI (*t* [62] = 8.33, *r* = 0.73 [0.48, 0.98], *p* < 0.001).

Younger women showed a trend towards lower SBPv compared to HRT‐women (*t* [63] = −1.69, *r* = −0.21 [−0.46, 0.04], *p* = 0.095), and higher vBRS compared to HRT‐women (*t* [63] = 1.72, *r* = 0.21 [−0.04, 0.46], *p* = 0.091) and nonHRT‐women (*t* [104] = 1.95, *r* = 0.19 [0.00, 0.38], *p* = 0.053). No other group differences reached statistical significance on mean and variability measures. Figures S1a,b and S2a,b depict mean differences between younger, nonHRT‐ and HRT‐women.

## 5 DISCUSSION

This study is the first investigation of age and sex differences in baroreflex‐mediated BP control focused on the vascular baroreflex limb. Consistent with the literature and our hypothesis, the vascular limb of the baroreflex was the primary mechanism of the age and sex differences we observed in BP control in adults. In particular, older women had significantly lower vBRS and higher TPR, SBP, and SBPv compared to younger women. In contrast, younger men had significantly higher vBRS, vBEI, TPR, and TPRv, along with lower SBP compared to older men. Additionally, among younger individuals, women had significantly lower vBEI, TPR, TPRv, SBP, and SBPv than men. This pattern reversed among older individuals, such that women had significantly higher vBEI, TPR, and SBP than men. Interestingly, younger men had higher vBRS, vBEI, TPR, TPRv, and SBPv than younger women and older men. When HRT was considered, HRT‐women had significantly lower SBP relative to nonHRT‐women and were similar to men on vBRS, vBEI, TPR, and SBP. In contrast, nonHRT‐women showed significantly higher vBEI, TPR, and SBP compared to men. Results align with prior work suggesting a modulating role of estrogen in the vascular system and highlight the need for future studies examining vascular baroreflex function. In summary, we provide additional evidence in support of the mechanistic role of the baroreflex in known age and sex differences in hypertension risk among adults.

### 5.1 Sex differences

It is well established that estrogen may play a critical role in the development of hypertension and cardiovascular outcomes. For instance, estrogen exerts genomic and nongenomic influences on the vascular endothelium via its anti‐inflammatory properties (Álvarez et al., 2002; Kubes et al., 1994; Toniolo et al., 2015; Trenti et al., 2018) and acts proactively in the vasculature, mediating endothelium‐derived nitric oxide production and bioavailability (Haynes et al., 2003), which has downstream effects on vasodilation, complementing vascular baroreflex control of BP (Cattaneo et al., 2017; Chakrabarti et al., 2008; Haynes et al., 2003; Lekontseva et al., 2010; Rossi et al., 2008; Sabbatini & Kararigas, 2020). As such, estrogen may, at least in part, explain age and sex differences in cardiovascular outcomes (Chakrabarti et al., 2008, 2014; Cignarella et al., 2010; Knowlton & Lee, 2012; Millett et al., 2018). In younger adults, women had lesser vBEI (i.e., reflexive TPR changes occurred 26% of the time), accompanied by relatively lower SBP and TPR compared to younger men. MSNA studies somewhat support this result, showing no relationship between MSNA and TPR or MSNA and BP in younger women and a positive relationship in younger men (Hart et al., 2011, 2014). However, our data show that with age, this pattern shifts, as older women showed greater vBEI alongside higher resting SBP and TPR compared to older men. These results are consistent with reports showing positive MSNA and TPR and MSNA and SBP relationships among older women and a steeper decline in endothelium‐dependent vasodilation among older women compared to similarly aged men (Hart et al., 2014; Taddei et al., 1996). Thus, whereas estrogen's protective effects in the vasculature appear more beneficial for younger women than men (Cattaneo et al., 2017; Keir et al., 2020; Kim‐Schulze et al., 1996; Taddei et al., 1996), our data and prior work indicate that these vascular benefits disappear with age, which coincides with declining circulating estrogen (Chakrabarti et al., 2008; Somani et al., 2019; Taddei et al., 1996; Toniolo et al., 2015; Vitale et al., 2008). Moreover, even when hypertension is managed, older women showed higher TPR‐mediated SBP, further supporting the idea that the underlying factors in elevated hypertension prevalence and risk of CV events in older women compared to men may be related to the loss of estrogen's protective effects in the vasculature (Millett et al., 2018; Rossi et al., 2008; Taddei et al., 1996) coupled with age‐related increases in the strength of the positive MSNA‐SBP relationship in older women (Keir et al., 2020). However, further studies are needed to evaluate vascular function in older adults without managed hypertension. Taken together, these findings are of particular importance as they might explain lower hypertension and CV risk among young women (Cutler et al., 2008).

Interestingly, despite differences in vBEI, vBRS was similar between women and men, regardless of age. Although speculative, these findings suggest a complex interplay between the strength (vBRS) and frequency (vBEI) of vascular responses to resting blood pressure oscillations. Namely, when BP oscillations are of lower magnitude, higher vBEI paired with higher vBRS may not be ideal, especially when TPR is higher; instead, although speculative, an optimal balance between the two may be more effective for regulating resting SBP oscillations. However, further research examining vascular baroreflex function is needed to elucidate these sex differences.

In contrast to earlier findings, it was somewhat surprising that men and women, regardless of age, were similar in cBRS. Prior work showed higher sensitivity in younger men than women in response to an intravenous ganglionic blockade (Christou et al., 2005). This inconsistency may be related to altered baroreflex sensitivity, which can occur when vasoactive drugs are injected intravenously (Parati et al., 2001; Parlow et al., 1995). Moreover, as cBRS in the preovulation phase differs (Minson et al., 2000) compared to when estrogen is higher in the early follicular and mid‐luteal phases (Tanaka et al., 2003), future studies on younger women should account for menstrual cycle information, as it was not available in our younger sample. Finally, cardiac and myocardial baroreflex measures may overlap, and factors affecting HR may confound both measures (i.e., ventricular preload) (Kenny et al., 1987; Reyes del Paso et al., 2017; van Lien et al., 2015). Thus, to further clarify our findings, future studies could include a measure of left ventricular contractility (e.g., pre‐ejection period; PEP).

### 5.2 Age differences

Vascular baroreflex‐mediated BP control also varied as a function of age, such that compared to their older counterparts, younger adults, regardless of sex, showed relatively more robust vascular responses to SBP oscillations accompanied by lower resting BP. Among younger women, lower BP and TPR were accompanied by greater vascular sensitivity, whereas older women exhibited higher TPR and likely should have shown higher vBEI. This suggests more effective TPR‐mediated BP control in younger women and reduced efficiency in older women. As previously stated, this is likely due to estrogen's anti‐inflammatory effects and protective role in vascular function in younger women (Chakrabarti et al., 2008, 2014; Sabbatini & Kararigas, 2020; Saitta et al., 2001; Taddei et al., 1996). For example, beta‐adrenergic‐mediated vasodilation may counterbalance alpha‐adrenergic vasoconstriction in younger but not older women (Hart et al., 2011; Joyner et al., 2014, 2016). Consequently, reduced estrogen levels in older women may contribute to differences in the effectiveness of the vascular baroreflex limb and known age‐related increases in hypertension and cardiovascular risk (Chakrabarti et al., 2014; Rossi et al., 2008; Somani et al., 2019; Taddei et al., 1996).

In younger men, higher TPR and TPRv were accompanied by reflexive TPR changes that occurred more often (i.e., around 32% of the time) compared to older men, suggesting effective vascular baroreflex function. However, our data and prior work suggest that sustained vascular stress observed in younger men (higher TPR and TPRv) may have long‐term effects on ventricular afterload and SV (Boutouyrie et al., 2021; Mitchell et al., 2007). Thus, although speculative, elevated cardiovascular risk in older men may be related to their higher SV being accompanied by less sensitivity and effectiveness in the myocardial limb. However, in men, endothelial function declines with age in a more variable way (Akishita et al., 2007; Celermajer et al., 1994; Hart et al., 2011). Thus, estrogen and testosterone may also contribute to elevated BP and hypertension risk among older men (Akishita et al., 2007; Empen et al., 2011; Harman et al., 2001; Kaur & Werstuck, 2021; Matsumoto, 2002; Newcomer et al., 2005). While estrogen's protective effects in men are not as well understood, emerging evidence suggests that low‐dose exogenous estrogen supplementation may lower BP and TPR, which has implications for reducing men's cardiovascular risk (Komesaroff et al., 2001; Saltiki et al., 2010).

Baroreflex function, including both sensitivity and effectiveness, was generally lower among older individuals, which may explain the increased risk of hypertension in this population. Vascular stress—characterized by chronic vasoconstriction and arterial stiffening—is strongly associated with aging and hypertension in both men and women (Gribbin et al., 1971; Mattace‐Raso et al., 2010; Mitchell et al., 2023; Monahan et al., 2001; Vlachopoulos et al., 2010). Since baroreceptors are sensitive to stretch and pressure, vascular stress may attenuate arterial distensibility (Avolio et al., 1985) and baroreflex sensitivity (Sapru & Wang, 1976), partially impairing the ability of these receptors to respond effectively to BP fluctuations (Kumada et al., 1990; McCubbin et al., 1956; Sapru & Wang, 1976; Thrasher, 2002), though some disagreement exists in the literature (see (Parati, 2005) for a review). These vascular changes, along with age‐related decreases in estrogen‐mediated vasodilation (Chakrabarti et al., 2008; Rudic et al., 1998) and vascular remodeling (Boutouyrie et al., 2021; Langille & Bendeck, 1990; Langille & O'Donnell, 1986; Mitchell et al., 2007; Monahan, 2007; Monahan et al., 2001), may contribute to declines in baroreflex function. However, future investigations should confirm whether the age‐related differences we observed persist in older adults without managed hypertension.

As spontaneous BPv may be more predictive of cardiovascular outcomes than 24‐h BPv (Dawson et al., 2000; Webb et al., 2018) and is linked with Alzheimer's disease risk (Lohman et al., 2024), our conflicting SBPv findings should be noted. Prior work has yielded equivocal results regarding age‐related differences in beat‐to‐beat BP variability (Fluckiger et al., 1999; Mancia et al., 1980; Parati et al., 1997). Our findings in women corroborate reports of age differences (Mancia et al., 1980; Parati et al., 1997), whereas our findings in men (i.e., no age effect) have also been reported (Fluckiger et al., 1999). Moreover, our younger sample showed sex differences in SBPv (i.e., lower SBPv in women) that contradict results from ambulatory BP studies (Thayer et al., 2016). However, there are well‐established circadian differences in ambulatory and resting BP (Mancia et al., 1980, 1983, 1986). Additionally, the extent to which short‐term (24‐h) and very short‐term (beat‐to‐beat) indicate attenuated reflexive arterial and cardio‐pulmonary control is still being investigated (Mancia et al., 1986; Parati et al., 2012). Thus, our conflicting results highlight the need for future studies that examine age and sex differences in baroreflex control of spontaneous BP and its variability.

### 5.3 HRT

The results of our exploratory analyses of menopausal hormone therapy and vascular function showed that women taking HRT had significantly lower SBP compared to women not taking HRT, as found in prior work (Fung et al., 2011). However, unlike previous reports, no differences in vasoconstriction or baroreflex sensitivity in the cardiac limb were observed (Gu et al., 2024; Herrington et al., 1994; Huikuri et al., 1996; Saitta et al., 2001). Additionally, compared to men, whereas nonHRT women had significantly higher SBP, TPR, and vBEI, these differences were slightly attenuated among HRT‐women. Although exploratory, our findings that younger women had similar vBEI with significantly lower SBP and TPR compared to nonHRT‐ and HRT‐women suggest that exogenous estrogen in older women may not offer the same vascular protective effects as endogenous estrogen in younger women, particularly for vascular baroreflex effectiveness or its underlying hemodynamics (Hodis et al., 2016; Joyner et al., 2016). Joyner et al. (2016) argue that healthy post‐menopausal women may lose the protective effects that offset alpha‐adrenergic vasoconstriction observed in younger women, contributing to a loss in beta‐adrenergically mediated vasodilation. We suggest that this might be mitigated at least in part by HRT, though the literature on the effects of HRT on MSNA is mixed (Nudy et al., 2019; Schierbeck et al., 2012; Vitale et al., 2008). For instance, a recent study found that 1 month of transdermal estradiol administration did not decrease resting MSNA in post‐menopausal women but did attenuate exercise‐related MSNA (Wenner et al., 2022). Interestingly, meta‐analyses and longitudinal evidence indicate that vasomotor symptoms (i.e., hot flushes) improve with HRT, which suggests it may have a promising role in vascular baroreflex function (Faubion et al., 2022; Santoro et al., 2016; Sarri et al., 2017). In summary, our results indicate that HRT may indeed be effective in lowering blood pressure, although minimal effects were observed on BP's underlying hemodynamics. However, given the controversy around the risks and benefits of HRT and our small sample of HRT‐women, our findings must be interpreted cautiously (Cho et al., 2023; Gu et al., 2024; Hodis et al., 2016; Rossouw et al., 2002, 2007). Moreover, the effectiveness of HRT on the baroreflex or the other underlying hemodynamics may vary as a function of the nature (e.g., exogenous estrogen and/or progesterone) and timing of HRT in relation to menopause onset (Nudy et al., 2019; Schierbeck et al., 2012; Vitale et al., 2008). Unfortunately, this information was not available in our study, so future studies with a larger sample are needed to determine the role of HRT in baroreflex control of BP.

## 6 LIMITATIONS AND FUTURE DIRECTIONS

As mentioned above, our results should be interpreted cautiously, as our study has several limitations. First, the time of day for BP measurements was less variable (early afternoon) in the older adults compared with the younger adults (any time between 9 am and 5 pm), which may have led to slight differences in SBP variability. While we had the younger adults refrain from exercise for 6 h, it is possible that they may have participated in intense physical activity, which may have influenced our results. Second, whereas some studies have suggested differences between BP measures taken in the sitting versus supine postures, most suggest that such differences are greater for DBP than SBP and decrease with age (Bartling et al., 2021; Lacruz et al., 2017; Privšek et al., 2018; Wei et al., 2008). Therefore, while it is unlikely that our findings were significantly influenced by the difference in posture between our two studies, future studies with BP measurements in the same posture are needed to replicate the present results. Additionally, as 62.4% (68 of 109) participants reported taking at least one cardiovascular medication, the extent to which participants' use of hypertension medications affected the underlying BP hemodynamics in the present study is unknown (Floras et al., 1988). Moreover, we were also underpowered to examine potential ethnic differences, which have been shown to have implications for CV outcomes (Willey et al., 2014; Williams et al., 2024; Young et al., 2020). Future studies should also consider including PEP to clarify the differential age effects observed in our study, as indices of the cardiac and myocardial baroreflex limbs may overlap (Kenny et al., 1987; van Lien et al., 2015). Finally, whereas prior reports show no differences in cBRS in the early follicular (EF) compared to the mid‐luteal (ML) menstrual cycle phases, differences were observed in the preovulation phase compared to the EF and ML phases (Minson et al., 2000). However, we had no menstrual cycle information for younger women (Tanaka et al., 2003).

## 7 CONCLUSION

Our findings provide evidence of age and sex differences in TPR‐mediated control of blood pressure (BP), as indicated by vascular baroreflex function, and suggest a potential role of estrogen in the well‐documented age‐ and sex‐related differences in cardiovascular disease (CVD) and hypertension risk (Benjamin et al., 2017; Cho et al., 2020; Cutler et al., 2008; McCarthy et al., 2014; Vitale et al., 2008; Whelton et al., 2018). These results underscore the critical role of baroreflex function in long‐term BP regulation and the development of hypertension (Bertinieri et al., 1988; Duschek & Reyes del Paso, 2007; Lohmeier, 2001; Lohmeier & Iliescu, 2015; Sleight, 1997, 2004). Although further research is needed to clarify the specific role of the vascular limb of the baroreflex, our findings align with meta‐analytic evidence suggesting that lifestyle modifications, such as diet and physical activity, can mitigate physiological changes in the vasculature and reduce risk in both women and men, regardless of age (Appel et al., 1997; Carlson et al., 2014; Edwards, de Caux, et al., 2022; Edwards, Wiles, & O'Driscoll, 2022; Inder et al., 2015; López‐Valenciano et al., 2019; Sacks et al., 2001; Whelton et al., 1997, 2018). In addition, our study may inform the creation of more tailored interventions for managing hypertension, potentially improving vascular function for those at elevated risk. For instance, a four‐month exercise training in middle‐aged hypertensives improved mean BP by 10 mmHg and reduced MSNA burst incidence by 40% compared to normotensives (Laterza et al., 2007). Additionally, a recent meta‐analysis demonstrated that isometric wall squats produced the greatest magnitude of reduction in SBP, DBP, MAP, and TPR, and improved BRS (Edwards, Wiles, & O'Driscoll, 2022). Importantly, given the relationship between CO and TPR to arterial pressure, these meta‐analytic findings suggest that BP reductions were TPR‐mediated. As found in prior meta‐analyses, isometric exercises have been found to be an effective non‐pharmacological antihypertensive treatment strategy regardless of hypertensive or medication status (Carlson et al., 2014; Edwards, de Caux, et al., 2022; Inder et al., 2015; López‐Valenciano et al., 2019). Thus, understanding individual differences in vascular baroreflex function using the sequence method among normotensives and managed hypertensives may enable clinicians to design personalized treatment strategies that account for individual physiological variations, leading to more effective approaches for reducing cardiovascular risk.

## FUNDING INFORMATION

This work was supported, in part, by the National Heart, Lung, and Blood Institute (R01HL126056) and the National Institute on Aging (R03AG063328). Darcianne Watanabe is supported by the National Science Foundation Graduate Research Fellowship Program under Grant No. DGE‐1839285. Any opinions, findings, and conclusions or recommendations expressed in this material are those of the author(s) and do not necessarily reflect the views of the National Science Foundation.

## CONFLICT OF INTEREST STATEMENT

None.

## ETHICS STATEMENT

The Institutional Review Board for both universities approved all procedures, and the studies were conducted in accordance with the ethical standards outlined in the 1964 Declaration of Helsinki.

## CONSENT TO PARTICIPATE

Written informed consent was obtained from all individual participants included in the study.

## Supporting information


**Figure S1.** Depict mean differences between younger women (sample one), women not on HRT (sample two), and HRT women (sample two) in systolic blood pressure in millimeters of mercury (SBP; a) and total peripheral resistance dynes per second per centimeter cubed per mmHg (TPR; b), with standard error bars.

**Figure S2.** Depict mean differences between younger women (sample one), women not on HRT (sample two), and HRT women (sample two) in baroreflex sensitivity in the vascular baroreflex limb in dynes per second per centimeter cubed per millimeters of mercury (vBRS; a) and baroreflex effectiveness in the vascular baroreflex limb in % (vBEI; b), with standard error bars.

## Data Availability

Data will be made available upon reasonable request.
