# Supplementary figures and images for: Age and sex differences in blood pressure regulation: A focus on the vascular baroreflex limb
Source: Physiol Rep. 2025 Jun 19;13(12):e70413. doi: 10.14814/phy2.70413 (PMC12179341; doi:10.14814/phy2.70413)

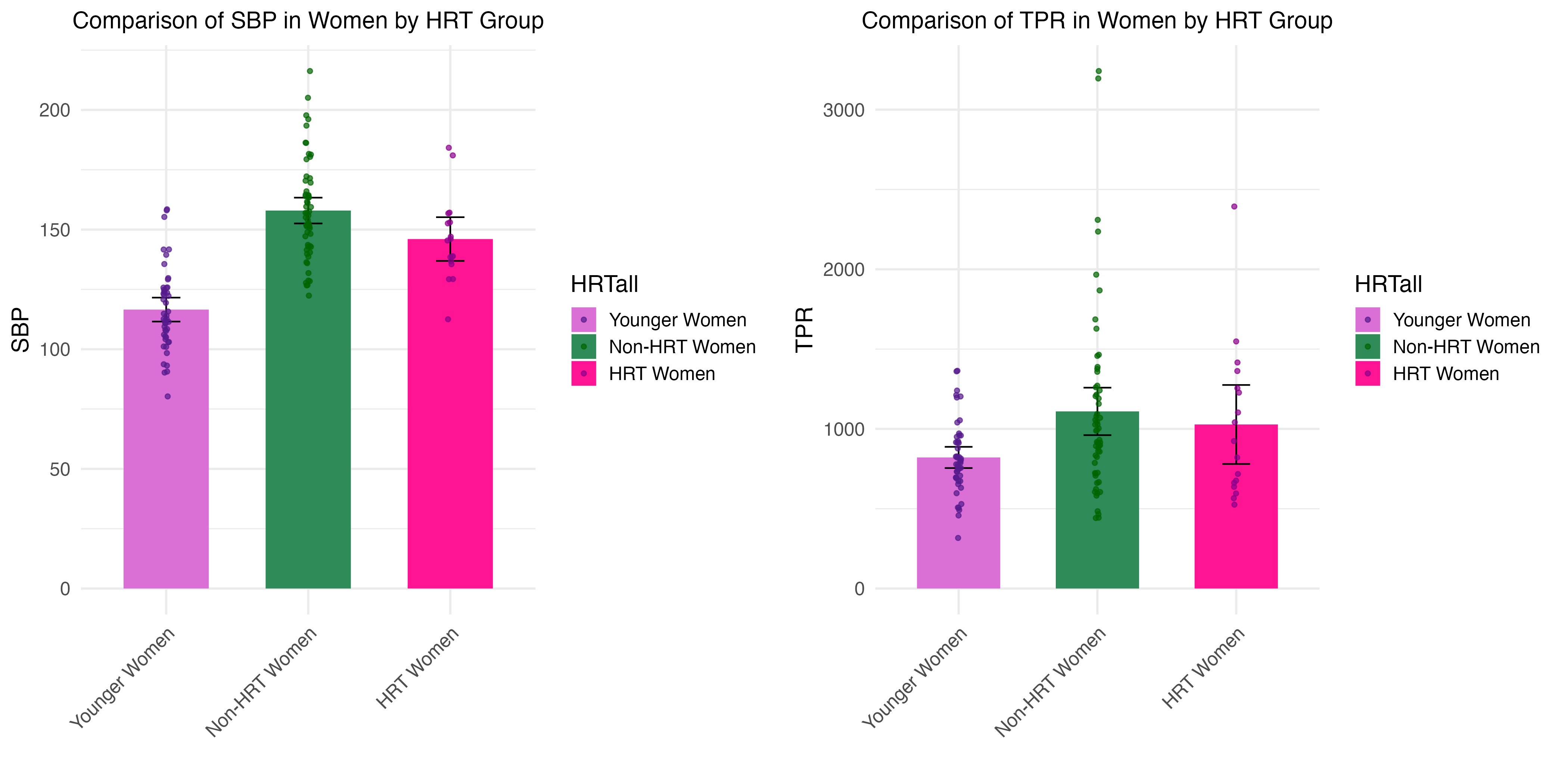

Supplement: Supplementary file 1 — Figure S1. Depict mean differences between younger women (sample one), women not on HRT (sample two), and HRT women (sample two) in systolic blood pressure in millimeters of mercury (SBP; a) and total peripheral resistance dynes per second per centimeter cubed per mmHg (TPR; b), with standard error bars. [file PHY2-13-e70413-s001.jpg]

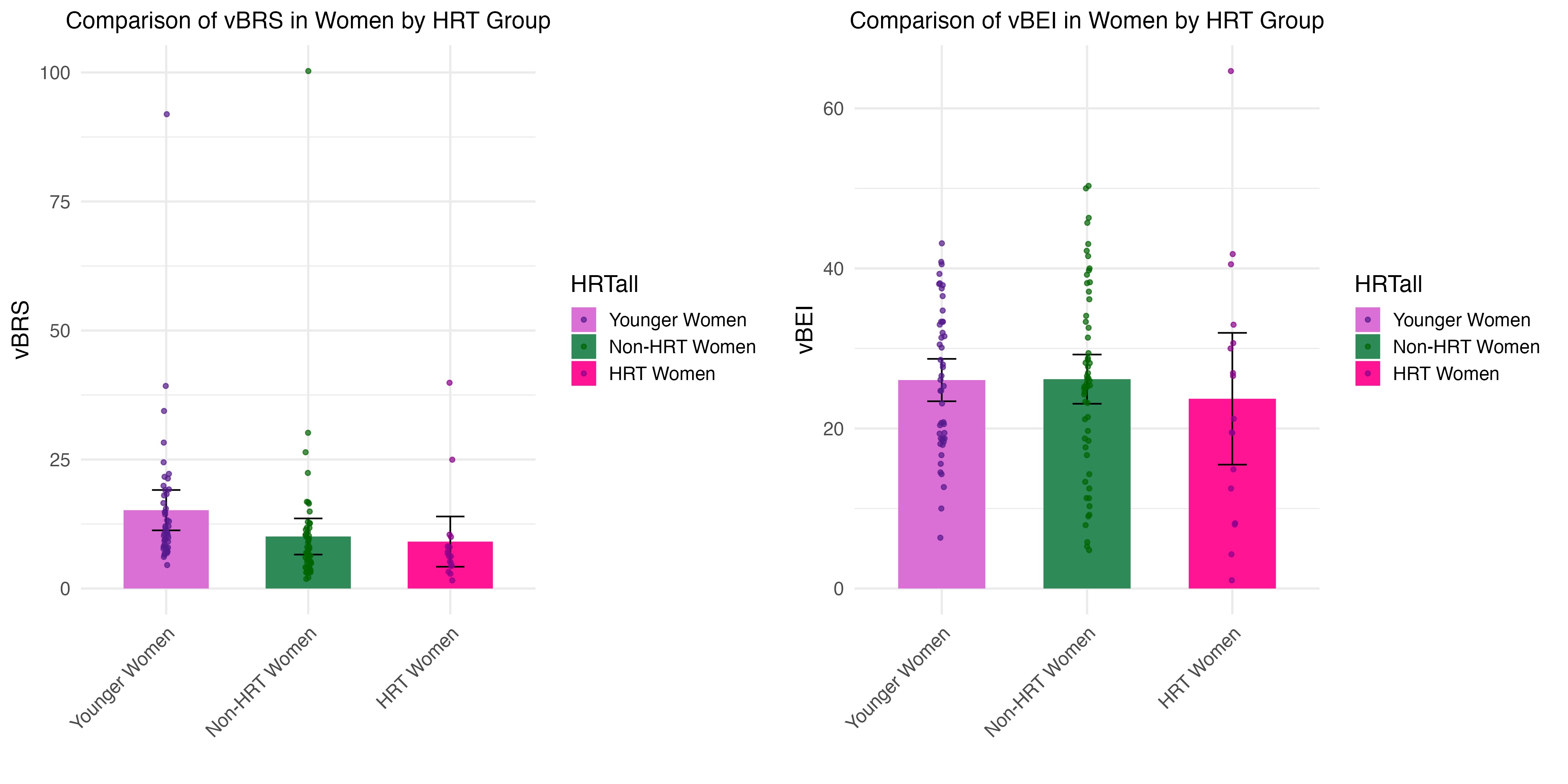

Supplement: Supplementary file 2 — Figure S2. Depict mean differences between younger women (sample one), women not on HRT (sample two), and HRT women (sample two) in baroreflex sensitivity in the vascular baroreflex limb in dynes per second per centimeter cubed per millimeters of mercury (vBRS; a) and baroreflex effectiveness in the vascular baroreflex limb in % (vBEI; b), with standard error bars. [file PHY2-13-e70413-s002.jpg]
